# Adaptive Fault-Tolerant Formation Control of Heterogeneous Multi-Agent Systems under Directed Communication Topology

**DOI:** 10.3390/s22166212

**Published:** 2022-08-18

**Authors:** Shangkun Liu, Bin Jiang, Zehui Mao, Yajie Ma

**Affiliations:** 1 College of Automation Engineering, Nanjing University of Aeronautics and Astronautics, Nanjing 211106, China; 2 Jiangsu Key Laboratory of Internet of Things and Control Technologies, Nanjing University of Aeronautics and Astronautics, Nanjing 211106, China

**Keywords:** fault-tolerant formation control, heterogeneous multi-agent systems, actuator faults, external disturbances, neural networks

## Abstract

This paper investigates the adaptive fault-tolerant formation control scheme for heterogeneous multi-agent systems consisting of unmanned aerial vehicles (UAVs) and unmanned surface vehicles (USVs) with actuator faults, parameter uncertainties and external disturbances under directed communication topology. Firstly, the dynamic models of UAVs and USVs are introduced, and a unified heterogeneous multi-agent system model with actuator faults is established. Then, a distributed fault-tolerant formation controller is proposed for the unified model of UAVs and USVs in the XY plane by using adaptive updating laws and radial basis function neural network. After that, a decentralized formation-tracking controller is designed for the altitude control system of UAVs. Based on the Lyapunov stability theory, it can be proved that the formation errors and tracking errors are uniformly ultimately bounded which means that the expected time-varying formation is achieved. Finally, a simulation study is given to demonstrate the effectiveness of the proposed scheme.

## 1. Introduction

In the last three decades, the formation control of multi-agent systems (MASs) has drawn lots of researchers’ attention in both theoretical research and practical applications, such as forest fire monitoring, power grid inspection, search and rescue, and so on [1,2,3,4,5,6]. The purpose of formation control is to form a specific formation to complete the tasks. One of the fundamental problem in MASs is consensus problem. Consensus problems of MASs have been investigated extensively and results have been achieved [7,8,9,10,11,12,13]. Based on the consensus theory, the formation control problem of MASs can be solved. In [14], the output-feedback formation control protocol of tracking a desired trajectory for a set of UAVs is developed. In [15], a distributed time-varying output formation control scheme is introduced for general linear MASs with directed topology. In [16], a distributed leader-following formation control for multiple quadrotors is studied by using nonsmooth backstepping method. In [17], a distributed relative position-based formation control by using backstepping approach is studied for leader–follower MASs.

However, the above results for formation control are used for homogeneous multi-agent systems. In the actual applications, for the heterogeneous multi-agent systems (HMASs) in which each agent may have different structures, dynamics and even the information perceptions and decision-making capabilities are considered. The time-varying formation-containment control for homogeneous and heterogeneous MASs is studied in [18]. In [19], the time-varying output formation-tracking scheme for linear HMASs is developed with directed topologies. In [20], the coherent formation control for HMASs is introduced. The distributed cooperative synchronization control problem for a networked HMASs is developed in [21]. The HMASs can achieve more complex and variable tasks through information interaction. For example, the HMASs consisting of multiple UAVs and USVs have a larger search radius and attack range, which is of great significance in both military and civil fields. Due to the heterogeneous characteristics of UAVs and USVs, as well as the influence of parameter uncertainties and external environment, the air–sea heterogeneous formation faces several important challenges which are shown as follows:

- UAVs and USVs differ notably in structure, model parameters, and state dimensions. Furthermore, the task changing is random, making it difficult to complete air–sea coordinated formation tasks.
- During the process of formation, the UAVs and USVs may inevitably be subjected to internal and external uncertainties, such as parameter uncertainties and external disturbances caused by modeling techniques and external environment.
- Unsatisfactory faults caused by a lot of damage to the actuator of the multiple UAVs and USVs system may affect the tracking performance.

In practical applications, faults may occur in the MASs. Faults may lead to system performance degradation, or even more serious consequences. Fault-tolerant control (FTC) is a useful method that has drawn wide attention [22,23,24,25]. In [26], a distributed adaptive leader-following formation control problem is studied for nonlinear second-order MASs in the presence of actuator faults. The distributed fault-tolerant time-varying formation control scheme for second-order MASs is presented subject to actuator faults in [27]. In [28], the active FTC problem is illustrated for the high-order HMASs with network disconnections and actuator faults. In [29], an adaptive FTC strategy by using a virtual actuator framework is presented for nonlinear HMASs with actuator faults. In addition, external disturbances and parameter uncertainties can also impact the performance of the systems. In [30], the time-varying formation tracking control scheme for the linear MASs is presented with external disturbances under directed graph. In [31], the time-varying anti-disturbance formation control scheme is introduced for nonlinear MASs under switched directed topologies. An adaptive tracking controller by adjusting the coupling weight is designed for the leader–follower linear MASs with external disturbances in [32]. In [33], an internal model approach is studied for the leader-following rendezvous with external disturbances and parameter uncertainties. During the process of formation, unmanned vehicles inevitably suffer from actuator faults, parameter uncertainties, and external disturbances. For example, due to modeling techniques and unpredictable marine environments, USVs are subject to internal and external uncertainties consisting of parameter uncertainties, nonparametric uncertainties, and external disturbances [34]. In addition, the actuator faults and uncertainties in a single vehicle can spread unevenly to neighboring vehicles through the directed communication topology in the process of formation. Hence, it is necessary to study the fault-tolerant formation control for HMASs consisting of multiple UAVs and USVs with parameter uncertainties and external disturbances. Although the research on FTC of MASs has achieved some results, the adaptive fault-tolerant formation control under directed communication topology for air-sea systems is still open, and needs to be further studied.

Motivated by the abovementioned results, this paper presents the adaptive fault-tolerant formation control scheme for multiple UAVs and USVs with actuator faults, parameter uncertainties, and external disturbances under directed communication topology. In order to handle the problems, a unified dynamic model of UAVs and USVs, which includes the XY plane and *Z* axis, are presented. To deal with the parameter uncertainties and external disturbances, a distributed fault-tolerant formation controller by combining adaptive control method and the radial basis function neural network is proposed in the XY plane and a decentralized formation-tracking controller is designed for the altitude control system of UAVs in the *Z* axis. The main contributions of this paper are summarized as

1. The adaptive fault-tolerant formation control is developed for the HMASs with parameter uncertainties, external disturbances, and actuator faults including loss of effectiveness and bias under directed communication topology. Some existing results have been researched for time-varying formation control, such as [16,34,35,36,37]. However, these results only consider the formation control of single vehicle or the HMASs consisting of a UAV and a USV. In this paper, the fault-tolerant time-varying formation for multiple UAVs and USVs can be achieved.
2. In order to handle parameter uncertainties and external disturbances, the adaptive control method and the radial basis function neural network (RBFNN) are combined to ensure that the formation errors and tracking errors of the closed-loop system are uniformly ultimately bounded.
3. Compared with the works [20,38], in which the height of each UAV is the same and time-invariant, this paper presents a decentralized tracking controller for the altitude control system of UAVs to track the reference signal to meet the requirements of practical tasks.

The rest of this paper is organized as follows. In Section 2, some preliminaries and problem formulation are given. In Section 3, the distributed adaptive fault-tolerant formation control scheme is introduced for multiple UAVs and USVs in the presence of actuator faults, parameter uncertainties and external disturbances. In Section 4, a decentralized formation tracking controller is proposed for the altitude control system of UAVs. In Section 5, a simulation study is given and some conclusions are drawn in Section 6.

**Notation** **1.** *∥∗∥ denotes the Euclidean norm. For a matrix A, $\lambda_{\max}\left(A\right)$ represents the maximum eigenvalue of matrix A, $\lambda_{2}\left(A\right)$ represents the minimum nonzero eigenvalue, and ${∥A∥}_{F}$ denotes the Frobenius norm of the matrix. Let 1 denote $1=\mathrm{col}\left\{1,\ldots ,1\right\}\in R^{N}$.*

## 2. Preliminaries and Problem Formulation

### 2.1. Graph Theory

Let G=(V,E) be a directed graph, in which V={1,2,…,N} represents the set of nodes and E⊆V×V represents the set of edges. The neighbour set of node *i* is denoted by $N_{i}=\left\{j\in V|\left(j,i\right)\in E\right\}$. If there exists a directed path between two arbitrary nodes, the graph G is strongly connected. $A=\left[a_{ij}\right]\in R^{N\times N}$ denotes the adjacency matrix of G, where $a_{ij}=1$ if (j,i)∈E and $a_{ij}=0$ otherwise. $D=\mathrm{diag}\{d_{1},d_{2},\ldots ,d_{N}\}$ denotes the degree matrix of G, where $d_{i}=\sum_{j=1}^{N}a_{ij}$. The Laplacian matrix of G is defined as L=D−A.

**Lemma** **1** ** ([39,40]).** *If the graph G is strongly connected, the following statements hold.*

- *There exists a vector $\varphi ={\left[\varphi_{1},\varphi_{2},\cdots ,\varphi_{N}\right]}^{⊤}$ with $\sum_{i=1}^{N}\varphi_{i}=1$ and $\varphi_{i}>0$, such that $\varphi^{⊤}L=0$.*
- *Define a symmetric matrix $\bar{L}=\Psi L+L^{⊤}\Psi$ with $\Psi =\mathrm{diag}\{\varphi_{1},\varphi_{2},\cdots ,\varphi_{N}\}$. Then $\bar{L}$ can be treated as Laplacian matrix associated with an undirected graph. Let $\varpi \left(t\right)\in R^{N\times 1}$, the following inequality holds*
$$\begin{array}{ccc} \min\varpi {\left(t\right)}^{⊤}\bar{L}\varpi \left(t\right)\; & > & \;\frac{\lambda_{2}\left(\bar{L}\right)}{N}\varpi {\left(t\right)}^{⊤}\varpi \left(t\right). \end{array}$$

### 2.2. Problem Formulation

In this subsection, the HMASs consisting of *M* UAVs and N−M USVs are considered. The dynamic models of the UAVs and USVs are given firstly. Based on them, a unified dynamic model for the HMASs is demonstrated. For convenience, let $\Pi_{1}=\left\{1,2,\ldots ,M\right\}$, $\Pi_{2}=\left\{M+1,M+2,\ldots ,N\right\}$ and $\Pi =\Pi_{1}⋃\Pi_{2}$.

**Figure 1:**
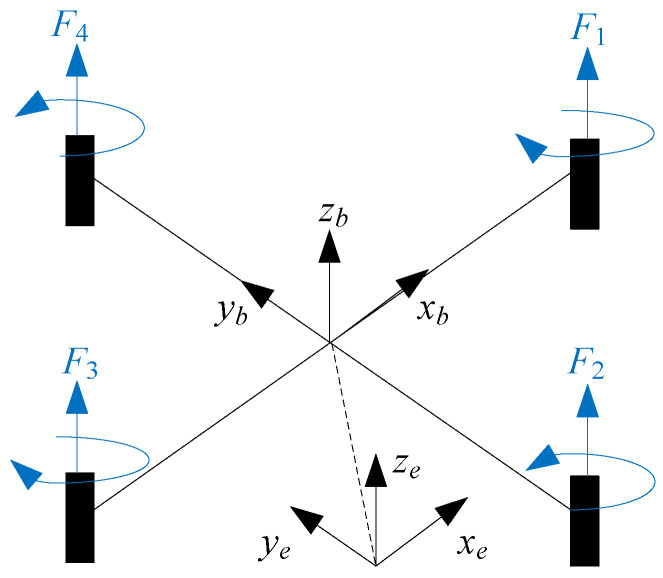
The structure of the quadrotor UAV.

Unmanned aerial vehicle model.The structure of the quadrotor UAV is shown in Figure 1. The dynamic model of the *i*-th ($i\in \Pi_{1}$) quadrotor UAV is given as [41]

(1) $$\begin{array}{c} \left\{\begin{array}{c} {\ddot{p}}_{aix}=\left(\cos\phi_{i}\sin\theta_{i}\cos\psi_{i}+\sin\phi_{i}\sin\psi_{i}\right)\frac{u_{pi}}{m_{ai}}-\frac{d_{ix}{\dot{p}}_{aix}}{m_{ai}}+\Delta_{aix},\\ {\ddot{p}}_{aiy}=\left(\cos\phi_{i}\sin\theta_{i}\sin\psi_{i}+\sin\phi_{i}\cos\psi_{i}\right)\frac{u_{pi}}{m_{ai}}-\frac{d_{iy}{\dot{p}}_{aiy}}{m_{ai}}+\Delta_{aiy},\\ {\ddot{p}}_{aiz}=\left(\cos\theta_{i}\cos\phi_{i}\right)\frac{u_{pi}}{m_{ai}}-\frac{d_{iz}{\dot{p}}_{aiz}}{m_{ai}}-g+\Delta_{aiz}, \end{array}\right. \end{array}$$

(2) $$\begin{array}{c} \left\{\begin{array}{c} {\ddot{\phi }}_{i}={\dot{\theta }}_{i}{\dot{\psi }}_{i}\frac{J_{ay}-J_{az}}{J_{ax}}-\frac{J_{ar}}{J_{ax}}{\dot{\theta }}_{i}{\bar{d}}_{i}+\frac{\tau_{\phi i}}{J_{ax}}-\frac{d_{i\phi }{\dot{\phi }}_{i}}{J_{ax}},\\ {\ddot{\theta }}_{i}={\dot{\phi }}_{i}{\dot{\psi }}_{i}\frac{J_{az}-J_{ax}}{J_{ay}}-\frac{J_{ar}}{J_{ay}}{\dot{\phi }}_{i}{\bar{d}}_{i}+\frac{\tau_{\theta i}}{J_{ay}}-\frac{d_{i\theta }{\dot{\theta }}_{i}}{J_{ay}},\\ {\ddot{\psi }}_{i}={\dot{\phi }}_{i}{\dot{\theta }}_{i}\frac{J_{ax}-J_{ay}}{J_{az}}+\frac{\tau_{\psi i}}{J_{az}}-\frac{d_{i\psi }{\dot{\psi }}_{i}}{J_{az}}, \end{array}\right. \end{array}$$

where ${\left[p_{aix},p_{aiy},p_{aiz}\right]}^{⊤}$ denotes the position state, ${\left[\phi_{i},\theta_{i},\psi_{i}\right]}^{⊤}$ denotes the attitude state, $u_{pi}$ denotes the control thrust of the quadrotor, $\tau_{\phi i}$, $\tau_{\theta i}$, $\tau_{\psi i}$ denote the three control torques of the quadrotor, $m_{ai}$ is the mass of the quadrotor UAV, *g* is the gravitational acceleration, ${\bar{d}}_{i}$ denotes the overall residual rotor angle, $d_{ix}$, $d_{iy}$, $d_{iz}$, $d_{i\phi }$, $d_{i\theta }$, $d_{i\psi }$ denote the translational drag coefficients, $J_{ax}$, $J_{ay}$, $J_{az}$ are the moments of the inertia, $J_{ar}$ is the moment of rotor’s inertia, $\Delta_{aix}$, $\Delta_{aiy}$, $\Delta_{aiz}$ denote the external disturbances encountered by the quadrotor UAV.

From the dynamic model (1) and (2), we can found that the motion of the quadrotor consists of translational dynamics with respect to positions and rotational dynamics with respect to angles. Our formation goal is to locate the center of mass in a predefined position, while the rotational dynamics can be stabilized separately. Hence, the position dynamics of the *i*-th ($i\in \Pi_{1}$) UAV based on (1) can be rewritten as

(3) $$\begin{array}{ccc} {\ddot{p}}_{ai}\; & = & \;g_{ai}u_{ai}+f_{ai}+\Delta_{ai}, \end{array}$$

where $p_{ai}={\left[p_{aix},p_{aiy},p_{aiz}\right]}^{⊤}$ denotes the position of the *i*-th UAV, $f_{ai}=[-d_{ix}{\dot{p}}_{aix}/m_{ai},$$-d_{iy}{\dot{p}}_{aiy}/m_{ai},-d_{iz}{\dot{p}}_{aiz}/m_{ai}{-g]}^{⊤}$, $g_{ai}=\mathrm{diag}\left\{1/m_{ai},1/m_{ai},1/m_{ai}\right\}$, $\Delta_{ai}={\left[\Delta_{aix},\Delta_{aiy},\Delta_{aiz}\right]}^{⊤}$, $u_{ai}={\left[u_{aix},u_{aiy},u_{aiz}\right]}^{⊤}$ is the new control signal which is given as [42]

$$\begin{array}{c} \left\{\begin{array}{c} u_{aix}=\left(\cos\phi_{i}\sin\theta_{i}\cos\psi_{i}+\sin\phi_{i}\sin\psi_{i}\right)u_{pi},\\ u_{aiy}=\left(\cos\phi_{i}\sin\theta_{i}\sin\psi_{i}+\sin\phi_{i}\cos\psi_{i}\right)u_{pi},\\ u_{aiz}=\left(\cos\theta_{i}\cos\phi_{i}\right)u_{pi}. \end{array}\right. \end{array}$$

Unmanned surface vehicle model. The kinematic and dynamic equation of the *i*-th ($i\in \Pi_{2}$) USV in the horizontal plane is described as [35]

(4) $$\begin{array}{c} \left\{\begin{array}{c} {\dot{x}}_{si}=\mu_{si}\cos\psi_{si}-\nu_{si}\sin\psi_{si},\\ {\dot{y}}_{si}=\mu_{si}\sin\psi_{si}+\nu_{si}\cos\psi_{si},\\ {\dot{\psi }}_{si}=r_{si}, \end{array}\right. \end{array}$$

(5) $$\begin{array}{c} \left\{\begin{array}{c} {\dot{\mu }}_{si}=f_{\mu si}\left(\alpha_{i}\right)+\frac{1}{m_{\mu si}}\left(\tau_{\mu si}^{f}+w_{\mu si}\right),\\ {\dot{\nu }}_{si}=f_{\nu si}\left(\alpha_{i}\right)+\frac{1}{m_{\nu si}}w_{\nu si},\\ {\dot{r}}_{si}=f_{rsi}\left(\alpha_{i}\right)+\frac{1}{m_{rsi}}\left(\tau_{rsi}^{f}+w_{rsi}\right), \end{array}\right. \end{array}$$

and

(6) $$\begin{array}{c} \left\{\begin{array}{c} f_{\mu si}\left(\alpha_{i}\right)=\frac{1}{m_{\mu si}}\left(m_{\nu si}\nu_{si}r_{si}-d_{\mu si}\mu_{si}-d_{\mu si1}\left|\mu_{si}\right|\mu_{si}\right),\\ f_{\nu si}\left(\alpha_{i}\right)=\frac{1}{m_{\nu si}}\left(-m_{\mu si}\mu_{si}r_{si}-d_{\nu si}\nu_{si}-d_{\nu si1}\left|\nu_{si}\right|\nu_{si}\right),\\ f_{rsi}\left(\alpha_{i}\right)=\frac{1}{m_{rsi}}\left(\left(m_{\mu si}-m_{\nu si}\right)\mu_{si}\nu_{si}-d_{rsi}r_{si}-d_{rsi1}\left|r_{si}\right|r_{si}\right), \end{array}\right. \end{array}$$

where ($x_{si}$, $y_{si}$) is the position of the *i*-th USV; $\psi_{si}$ is the yaw angle of the *i*-th USV; $\alpha_{i}={\left[\mu_{si},\nu_{si},r_{si}\right]}^{⊤}$ are the surge, sway, and yaw velocity, respectively; $m_{\mu si}$, $m_{\nu si}$, $m_{rusi}$ are the inertial mass; $f_{\mu si}\left(\alpha_{i}\right)$, $f_{\nu si}\left(\alpha_{i}\right)$, $f_{rsi}\left(\alpha_{i}\right)$ are the nonlinear dynamics consisting of the unmodeled hydrodynamics and Coriolis forces; $\tau_{\mu si}^{f}$ and $\tau_{rsi}^{f}$ are the surge force and the yaw moment; and $w_{\mu si}$, $w_{\nu si}$, $w_{rsi}$ are the bounded disturbances caused by waves, wind, and ocean currents.

**Figure 2:**
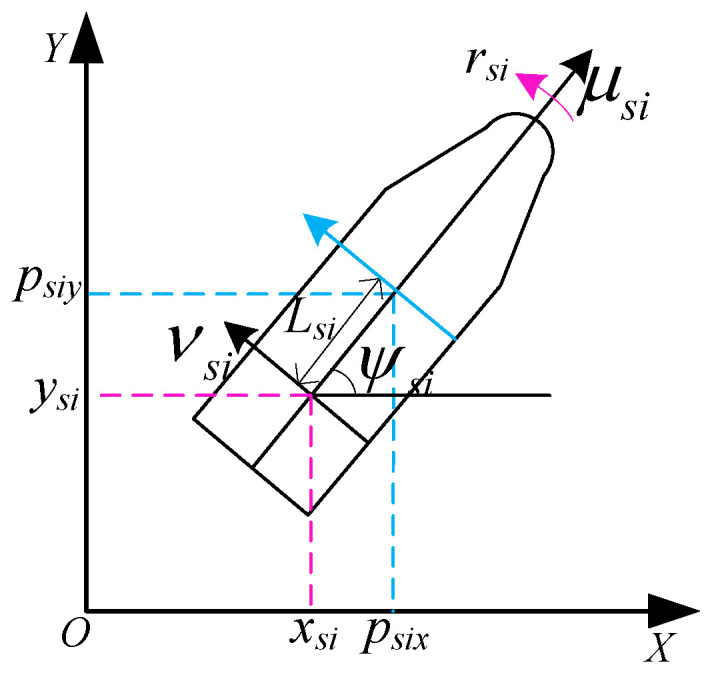
The motion model of USV.

Since the motion model of the USVs described by (4) and (5) is underactuated, a hand position approach is used to deal with it. We define the front point $\left(p_{six},p_{siy}\right)$ of the USVs as the hand point which can be formulated as

(7) $$\begin{array}{c} \left\{\begin{array}{c} p_{six}=x_{si}+L_{si}\cos\psi_{si},\\ p_{siy}=y_{si}+L_{si}\sin\psi_{si}, \end{array}\right. \end{array}$$

where $L_{si}$ is the distance between the actual position $\left(x_{si},y_{si}\right)$ and the new defined hand point $\left(p_{six},p_{siy}\right)$, which is shown in Figure 2.

By taking the second derivative of (7), one can obtain

(8) $$\begin{array}{c} \left\{\begin{array}{cc} {\ddot{p}}_{six}= & \;{\dot{\mu }}_{si}\cos\psi_{si}-\left({\dot{\nu }}_{si}+L_{si}{\dot{r}}_{si}\right)\sin\psi_{si}-\mu_{si}r_{si}\sin\psi_{si}\\ & \;-\left(\nu_{si}r_{si}+L_{si}r_{si}^{2}\right)\cos\psi_{si},\\ {\ddot{p}}_{siy}= & \;{\dot{\mu }}_{si}\sin\psi_{si}-\left({\dot{\nu }}_{si}+L_{si}{\dot{r}}_{si}\right)\cos\psi_{si}+\mu_{si}r_{si}\cos\psi_{si}\\ & \;-\left(\nu_{si}r_{si}+L_{si}r_{si}^{2}\right)\sin\psi_{si}, \end{array}\right. \end{array}$$

Substituting (6) into (8) yields that

(9) $$\begin{array}{c} \left\{\begin{array}{c} {\ddot{p}}_{six}=f_{six}\left(\beta \right)+\frac{\cos\psi_{si}}{m_{\mu si}}\tau_{\mu }^{f}-\frac{L_{si}\sin\psi_{si}}{m_{rsi}}\tau_{r}^{f}+w_{dix},\\ {\ddot{p}}_{siy}=f_{siy}\left(\beta \right)+\frac{\sin\psi_{si}}{m_{\mu si}}\tau_{\mu }^{f}+\frac{L_{si}\cos\psi_{si}}{m_{rsi}}\tau_{r}^{f}+w_{diy}, \end{array}\right. \end{array}$$

where

(10) $$\begin{array}{c} \left\{\begin{array}{cc} f_{six}\left(\beta \right)= & \;f_{\mu }\left(\alpha \right)\cos\psi_{si}-\left(f_{\nu }\left(\alpha \right)+L_{si}f_{r}\left(\alpha \right)\right)\sin\psi_{si}\\ & \;-\mu_{si}r_{si}\sin\psi_{si}-\left(\nu_{si}r_{si}+L_{si}r_{si}^{2}\right)\cos\psi_{si},\\ f_{siy}\left(\beta \right)= & \;f_{\mu }\left(\alpha \right)\sin\psi_{si}+\left(f_{\nu }\left(\alpha \right)+L_{si}f_{r}\left(\alpha \right)\right)\cos\psi_{si}\\ & \;+\mu_{si}r_{si}\cos\psi_{si}-\left(\nu_{si}r_{si}+L_{si}r_{si}^{2}\right)\sin\psi_{si}, \end{array}\right. \end{array}$$

and

(11) $$\begin{array}{c} \left\{\begin{array}{c} w_{dix}\left(\beta \right)=\frac{w_{\mu si}}{m_{\mu si}}\cos\psi_{si}-\left(\frac{w_{\nu si}}{m_{\mu si}}+\frac{L_{si}w_{rsi}}{m_{rsi}}\right)\sin\psi_{si},\\ w_{diy}\left(\beta \right)=\frac{w_{\mu si}}{m_{\mu si}}\sin\psi_{si}+\left(\frac{w_{\nu si}}{m_{\mu si}}+\frac{L_{si}w_{rsi}}{m_{rsi}}\right)\cos\psi_{si}, \end{array}\right. \end{array}$$

with $\beta =[\mu_{si},\nu_{si},r_{si},\psi_{si}]$.

Based on (9), the position dynamics of the *i*-th ($i\in \Pi_{2}$) USV can be described as

(12) $$\begin{array}{ccc} {\ddot{p}}_{si}\; & = & \;f_{sixy}+\Omega_{si}\left(\psi_{si}\right)\varpi_{si}u_{si}+w_{dixy}, \end{array}$$

where $p_{si}={\left[p_{six},p_{siy}\right]}^{⊤}$ is the position of the *i*-th ($i\in \Pi_{2}$) USV, $f_{sixy}={\left[f_{six},f_{siy}\right]}^{⊤}$, $\Omega_{si}\left(\psi_{si}\right)=\left[\cos\psi_{si},-\sin\psi_{si};\sin\psi_{si},\cos\psi_{si}\right]$, $u_{si}={\left[\tau_{\mu },\tau_{r}\right]}^{⊤}$, $\varpi_{si}=\mathrm{diag}\left\{1/m_{\mu si},L_{si}/m_{rsi}\right\}$, $w_{dixy}={\left[w_{dix},w_{diy}\right]}^{⊤}$.

Actuator fault model.The actuator fault model for the *h*-th actuator of the *i*-th (i∈Π) HMASs is given as

(13) $$\begin{array}{ccc} u_{ih}^{F}\; & = & \;\rho_{ih}u_{ih}+u_{ihb}, \end{array}$$

where $u_{ih}^{F}$ is the actual actuation input, $u_{ih}$ is the applied control signal to be designed, $0<\rho_{ih}\leq 1$ is the unknown effectiveness factor and $u_{ihb}$ represents the unknown bias. For the actuator fault model (13), $\rho_{ih}=1$ and $u_{ihb}=0$ denote that there is no fault; $0<\rho_{ih}<1$ and $u_{ihb}=0$ denote the loss of effectiveness fault; $\rho_{ih}=0$ and $u_{ihb}\neq 0$ denote bias fault; $0<\rho_{ih}<1$ and $u_{ihb}\neq 0$ denote both loss of effectiveness and bias faults.

From (13), the fault model for all actuators can be described as

(14) $$\begin{array}{ccc} u_{i}^{F}\; & = & \;\rho_{i}u_{i}+u_{ib},\;\;\;i\in \Pi , \end{array}$$

where $\rho_{i}=\mathrm{diag}\left\{\rho_{i1},\rho_{i2},\ldots ,\rho_{in}\right\}$ and $u_{ib}={\left[u_{ib1},u_{ib2},\ldots ,u_{ibn}\right]}^{⊤}$ with *n* representing the dimension of the control input signal.

Unified model. The UAV model with actuator faults (14) in the XY plane can be rewritten as

(15) $$\begin{array}{c} \left\{\begin{array}{cc} {\dot{x}}_{ai1}= & \;x_{ai2},\\ {\dot{x}}_{ai2}= & \;f_{aixy}+g_{aixy}\left(\rho_{ai}u_{aixy}+u_{aib}\right)+\Delta_{aixy}\\ & \;=F_{aixy}+G_{aixy}u_{aixy}+\Delta_{aixy}, \end{array}\right. \end{array}$$

where $x_{ai1}={\left[p_{aix},p_{aiy}\right]}^{⊤}$ is the position of the *i*-th UAV in the XY plane, $u_{aixy}={\left[u_{aix},u_{aiy}\right]}^{⊤}$, $g_{aixy}=\mathrm{diag}\left\{1/m_{ai},1/m_{ai}\right\}$, $f_{aixy}={\left[-d_{ix}{\dot{p}}_{aix}/m_{ai},-d_{iy}{\dot{p}}_{aiy}/m_{ai}\right]}^{⊤}$, $F_{aixy}=f_{aixy}+g_{aixy}u_{aib}$, $G_{aixy}=g_{aixy}\rho_{ai}$, $\rho_{ai}=\mathrm{diag}\left\{\rho_{aix},\rho_{aiy}\right\}$ denotes the effectiveness factor, $u_{aib}={\left[u_{aibx},u_{aiby}\right]}^{⊤}$ denotes the bias, $\Delta_{aixy}={\left[\Delta_{aix},\Delta_{aiy}\right]}^{⊤}$.

Similarly, the UAV model with actuator faults (14) in the *Z* axis can be rewritten as

(16) $$\begin{array}{c} \left\{\begin{array}{cc} {\dot{p}}_{aiz}= & \;v_{aiz},\\ {\dot{v}}_{aiz}= & \;f_{aiz}+\frac{1}{m_{ai}}\left(\rho_{aiz}u_{aiz}+u_{aibz}\right)+\Delta_{aiz}\\ & \;=F_{aiz}+G_{aiz}u_{aiz}+\Delta_{aiz}, \end{array}\right. \end{array}$$

where $p_{aiz}$ is the altitude of the *i*-th UAV, $v_{aiz}$ is the velocity of the *i*-th UAV in the *Z* axis, $f_{aiz}=-d_{iz}{\dot{p}}_{aiz}/m_{ai}-g$, $\rho_{aiz}$ is the effectiveness factor, $u_{aibz}$ is the bias, $F_{aiz}=f_{aiz}+u_{aibz}/m_{ai}$, $G_{aiz}=\rho_{ai}/m_{ai}$.

The USV model with actuator faults (14) can be rewritten as

(17) $$\begin{array}{c} \left\{\begin{array}{cc} {\dot{x}}_{si1}= & \;x_{si2},\\ {\dot{x}}_{si2}= & \;f_{sixy}+g_{si}\left(\rho_{si}u_{si}+u_{sib}\right)+w_{dixy}\\ & \;=F_{si}+G_{si}u_{si}+w_{dixy}, \end{array}\right. \end{array}$$

where $x_{si1}=p_{si}$, $\rho_{si}=\mathrm{diag}\left\{\rho_{six},\rho_{siy}\right\}$ is the effectiveness factor, $u_{sib}={\left[u_{sibx},u_{siby}\right]}^{⊤}$ is the bias, $g_{si}=\Omega_{si}\left(\psi_{si}\right)\varpi_{si}$, $F_{si}=f_{sixy}+g_{si}u_{sib}$, $G_{si}=g_{si}\rho_{si}$.

Combining (15) and (17), the unified model of UAVs and USVs in the XY plane can be obtained

(18) $$\begin{array}{c} \left\{\begin{array}{c} {\dot{x}}_{i1}=x_{i2},\\ {\dot{x}}_{i2}=F_{xi}+G_{xi}u_{xi}+\Delta_{xi}, \end{array}\right. \end{array}$$

where $x_{i1}=x_{ai1}\in R^{2}$, $x_{i2}=x_{ai2}\in R^{2}$ are the position and velocity of the *i*-th ($i\in \Pi_{1}$) UAV in the XY plane, $F_{xi}=F_{aixy}$, $G_{xi}=G_{aixy}$, $\Delta_{xi}=\Delta_{aixy}$, $u_{xi}=u_{aixy}$; $x_{i1}=x_{si1}\in R^{2}$, $x_{i2}=x_{si2}\in R^{2}$ are the position and velocity of the *i*-th ($i\in \Pi_{2}$) USV, $F_{xi}=F_{si}$, $G_{xi}=G_{si}$, $\Delta_{xi}=w_{dixy}$, $u_{xi}=u_{si}$.

Hence, we are under the situation to design the fault-tolerant time-varying formation control scheme for the HMASs consisting of multiple UAVs and USVs in the presence of unknown actuator faults, parameter uncertainties and external disturbances under directed topology.

**Remark** **1.** *In the inertial frame O−XYZ, the USVs only move in the XY plane. The UAVs move in three-dimensional space, but their motion in the Z axis can be decoupled from that in the XY plane. Hence, the height of the UAVs can be controlled independently. Therefore, the fault-tolerant time-varying formation control for multiple UAVs and USVs is considered in the XY plane. At the same time, the formation-tracking controller is designed for the UAVs so that the height of the UAVs can track the reference signal. In this way, the fault-tolerant time-varying formation control for multiple UAVs and USVs can be achieved.*

**Assumption** **1.** *The effectiveness factor $\rho_{ih}$ and bias $u_{ihb}$ are unknown but bounded. There exist positive constants ${\underline{\rho }}_{ih}$, ${\bar{\rho }}_{ih}$ and ${\bar{u}}_{ihb}$ satisfying $0<{\underline{\rho }}_{ih}\leq \rho_{ih}\leq {\bar{\rho }}_{ih}\leq 1$ and $u_{ihb}\leq {\bar{u}}_{ihb}$, respectively.*

**Assumption** **2.** *The aerodynamic drag coefficients $d_{ix}$, $d_{iy}$ and $d_{iz}$ are unknown but bounded.*

**Assumption** **3.** *The external disturbances $\Delta_{aix}$, $\Delta_{aiy}$, $\Delta_{aiz}$ encountered by the quadrotor UAV are bounded and satisfying $∥\Delta_{aix}∥\leq {\bar{\Delta }}_{aix}$, $∥\Delta_{aiy}∥\leq {\bar{\Delta }}_{aiy}$, $∥\Delta_{aiz}∥\leq {\bar{\Delta }}_{aiz}$, where ${\bar{\Delta }}_{aix}$, ${\bar{\Delta }}_{aiy}$ and ${\bar{\Delta }}_{aiz}$ are unknown positive constants.*

**Assumption** **4.** *The external disturbances $w_{\mu si}$, $w_{\nu si}$, $w_{rsi}$ encountered by the USV are bounded and satisfying $∥w_{\mu si}∥\leq {\bar{w}}_{\mu si}$, $∥w_{\nu si}∥\leq {\bar{w}}_{\nu si}$, $∥w_{rsi}∥\leq {\bar{w}}_{rsi}$, where ${\bar{w}}_{\mu si}$, ${\bar{w}}_{\nu si}$ and ${\bar{w}}_{rsi}$ are unknown positive constants.*

**Assumption** **5.** *In (4), the sway velocity $\nu_{si}$ of the underactuated USV is passively-bounded.*

**Remark** **2.** *Assumption 1 is standard to handle the actuator faults in the existing literature [43,44]. Due to the unpredictable aerodynamics produced by the complex operation environment, it is difficult to obtain accurate system parameters, so Assumption 2 is reasonable and realistic. Since UAVs and USVs will not be used under extreme whether conditions, Assumptions 3 and 4 are also reasonable. Superficially, Assumption 5 seems to be restrictive. However, it is easy to verify that Assumption 5 is always satisfied in most practical applications of the USVs [45].*

### 2.3. Radial Basis Function Neural Network

Since the radial basis function neural network can approximate any continuous function with arbitrary precision, in this paper, the RBFNN is used to approximate the nonlinear function. The RBFNN can be expressed as

(19) $$\begin{array}{ccc} f(Z)\; & = & \;\theta^{*⊤}w\left(Z\right)+\epsilon \left(Z\right),\;\;Z\in \Omega_{Z}, \end{array}$$

where f(Z) is a smooth nonlinear function, $Z={\left[Z_{1},Z_{2},\ldots ,Z_{n}\right]}^{⊤}\in \Omega_{Z}\subset R^{n}$ denotes the input vector, $w\left(Z\right)={\left[w_{1}\left(Z\right),w_{2}\left(Z\right),\ldots ,w_{p}\left(Z\right)\right]}^{⊤}\in R^{p}$ is the basis function vector, *p* is the number of the RBFNN nodes. ϵ(Z) is the approximation error, bounded by $∥\epsilon \left(Z\right)∥\leq \bar{\epsilon }$. $\theta^{*}={\left[\theta_{1},\theta_{2},\ldots ,\theta_{p}\right]}^{⊤}\in R^{p}$ is the ideal weighting vector, which is denoted as

(20) $$\begin{array}{ccc} \theta^{*}\; & = & \;\arg\min_{\theta \in R^{p}}\{\underset{Z\in \Omega_{Z}}{\sup}|f\left(Z\right)-\theta^{⊤}w\left(Z\right)\left|\right\}, \end{array}$$

where θ denotes the weighting vector.

### 2.4. Control Objective

The control objective of this paper is to design an adaptive fault-tolerant formation control scheme for multiple UAVs and USVs (16) and (18) to achieve the time-varying formation of the HMASs in the presence of unknown actuator faults, parameter uncertainties and external disturbances under directed topology, that is, for any given bounded initial states,

$$\begin{array}{c} \left\{\begin{array}{c} \lim_{t\to \infty }∥\left(x_{i1}-h_{i1}\right)-\left(x_{j1}-h_{j1}\right)∥\leq \chi_{1},\;\lim_{t\to \infty }∥\left(x_{i2}-h_{i2}\right)∥\leq \chi_{2},\;i\in \Pi ,\\ \lim_{t\to \infty }∥p_{aiz}-c_{ip}∥\leq \chi_{3},\;\lim_{t\to \infty }∥v_{aiz}-c_{iv}∥\leq \chi_{4},\;i\in \Pi_{1}, \end{array}\right. \end{array}$$

where $\chi_{1}$, $\chi_{2}$, $\chi_{3}$ and $\chi_{4}$ are small enough positive constants, $h\left(t\right)={\left[h_{1}\left(t\right),h_{2}\left(t\right),\ldots ,h_{N}\left(t\right)\right]}^{⊤}$ is the desired time-varying formation with $h_{i}\left(t\right)={\left[h_{i1}\left(t\right),h_{i2}\left(t\right)\right]}^{⊤}$ being piecewise continuously differentiable. $h_{i1}\left(t\right)$ and $h_{i2}\left(t\right)$ are the position and velocity, respectively. $c_{ip}$ and $c_{iv}$ are the desired height and velocity reference signal, respectively.

## 3. Distributed Fault-Tolerant Formation Control Scheme and Performance Analysis

In this section, a distributed fault-tolerant formation control scheme is designed to achieve the time-varying formation control of HMASs composed of UAVs and USVs with actuator faults, parameter uncertainties and external disturbances under directed topology in the XY plane.

### 3.1. Distributed Fault-Tolerant Formation Controller Design

Define a synchronization formation error signal for each vehicle as

(21) $$\begin{array}{ccc} e_{i}\; & = & \;\sum_{j=1}^{N}a_{ij}\left(\left(x_{i1}-h_{i1}\right)-\left(x_{j1}-h_{j2}\right)\right)+x_{i2}-h_{i2},\;\;\;i\in \Pi . \end{array}$$

Let $z_{i}=x_{i1}-h_{i1}$ and $s_{i}=x_{i2}-h_{i2}$. Then the time derivative of $z_{i}$ and $s_{i}$ can be written as

(22) $$\begin{array}{ccc} {\dot{z}}_{i}\; & = & \;x_{i2}+h_{i2}-{\dot{h}}_{i1}, \end{array}$$

(23) $$\begin{array}{ccc} {\dot{s}}_{i}\; & = & \;F_{xi}+G_{xi}u_{xi}+\Delta_{xi}-{\dot{h}}_{i2}. \end{array}$$

Let ${\tilde{z}}_{i}=z_{i}-\sum_{j=1}^{N}\varphi_{j}z_{j}$, where $\varphi_{j}$ is given in Lemma 1. Let $\tilde{z}={\left[{\tilde{z}}_{1},{\tilde{z}}_{2},\ldots ,{\tilde{z}}_{N}\right]}^{⊤}$, $e={\left[e_{1},e_{2},\ldots ,e_{N}\right]}^{⊤}$. If the feasibility condition $h_{i2}-{\dot{h}}_{i1}=0$ is satisfied, then the HMASs (22) and (23) based on the definitions of $\tilde{z}$ and *e* can be written as

(24) $$\begin{array}{ccc} \dot{\tilde{z}}\; & = & \;\left(I_{N}-1\varphi^{⊤}\right)e-L\tilde{z}, \end{array}$$

(25) $$\begin{array}{ccc} \dot{e}\; & = & \;Le-L^{2}\tilde{z}+F_{x}+G_{x}u_{x}+\Delta_{x}-{\dot{h}}_{2}, \end{array}$$

where $F_{x}={\left[F_{x1},F_{x2},\ldots ,F_{xN}\right]}^{⊤}$, $G_{x}=\mathrm{diag}\left\{G_{x1},G_{x2},\ldots ,G_{xN}\right\}$, ${\dot{h}}_{2}={\left[{\dot{h}}_{12},{\dot{h}}_{22},\ldots ,{\dot{h}}_{N2}\right]}^{⊤}$, $\Delta_{x}={\left[\Delta_{x1},\Delta_{x2},\ldots ,\Delta_{xN}\right]}^{⊤}$, $u_{x}={\left[u_{x1},u_{x2},\ldots ,u_{xN}\right]}^{⊤}$.

The control input of the HMASs and the adaptive laws are developed as

(26) $$\begin{array}{ccc} u_{xi}\; & = & \;{\hat{G}}_{xi}^{-1}\left(-\delta_{i}e_{i}-\frac{e_{i}{\hat{\kappa }}_{i11}\omega_{i1}^{⊤}\omega_{i1}}{2\varsigma_{i1}^{2}}-\frac{e_{i}{\hat{\kappa }}_{i12}}{2\varsigma_{i2}^{2}}+{\dot{h}}_{i2}\right), \end{array}$$

(27) $$\begin{array}{ccc} {\dot{\hat{\kappa }}}_{i11}\; & = & \;\iota_{11}\left(-k_{11}{\hat{\kappa }}_{i11}+\frac{e_{i}^{⊤}e_{i}\omega_{i1}^{⊤}\omega_{i1}}{2\varsigma_{i1}^{2}}\right), \end{array}$$

(28) $$\begin{array}{ccc} {\dot{\hat{\kappa }}}_{i12}\; & = & \;\iota_{12}\left(-k_{12}{\hat{\kappa }}_{i12}+\frac{e_{i}^{⊤}e_{i}}{2\varsigma_{i2}^{2}}\right), \end{array}$$

(29) $$\begin{array}{ccc} {\dot{\hat{G}}}_{xi}\; & = & \;{\mathrm{Proj}}_{\left[{\underline{G}}_{xi},{\bar{G}}_{xi}\right]}\left\{P\right\}=\left\{\begin{array}{cc} 0, & \mathrm{if}\;{\hat{G}}_{xi}={\bar{G}}_{xi}\;\mathrm{and}\;P\geq 0\;\\ & \mathrm{or}\;{\hat{G}}_{xi}={\underline{G}}_{xi}\;\mathrm{and}\;P\leq 0,\\ P, & \mathrm{otherwise}, \end{array}\right. \end{array}$$

where $P=\iota_{13}\left(-k_{13}{\hat{G}}_{xi}+e_{i}u_{xi}\right)$, ${\underline{G}}_{xi}$ and ${\bar{G}}_{xi}$ are the lower bound and upper bound of the parameter $G_{xi}$, respectively, $\delta_{i}$, $k_{11}$, $k_{12}$, $k_{13}$, $\iota_{11}$, $\iota_{12}$, $\iota_{13}$, $\varsigma_{i1}$, $\varsigma_{i2}$ are positive parameters to be designed.

**Figure 3:**
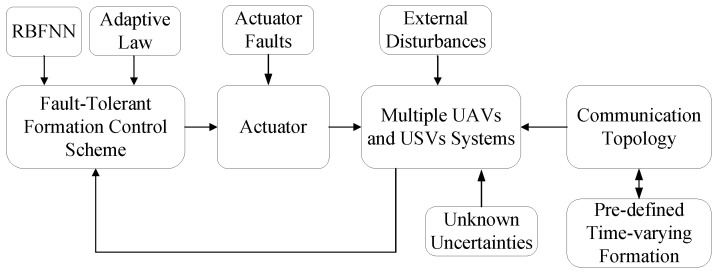
Schematic of the control system.

The schematic of the control system is illustrated in Figure 3.

### 3.2. Performance Analysis

The performance of the time-varying formation error system is given as follows.

**Theorem** **1.** *Consider a heterogeneous multi-agent system (18). Suppose that Assumptions 1–5 hold and the feasibility condition $h_{i2}-{\dot{h}}_{i1}=0$ of the time-varying formation is satisfied, the fault-tolerant control scheme is designed as (26) and the adaptive laws are developed as (27)–(29), then the time-varying formation errors $\tilde{z}$ and e in the XY plane are uniformly ultimately bounded.*

**Proof** **of Theorem 1.** Consider the following Lyapunov candidate function

(30) $$\begin{array}{ccc} V\; & = & \;{\tilde{z}}^{⊤}\Psi \tilde{z}+\frac{1}{2}e^{⊤}e+\sum_{i=1}^{N}\frac{{\tilde{\kappa }}_{i11}^{2}}{2\iota_{11}}+\sum_{i=1}^{N}\frac{{\tilde{\kappa }}_{i12}^{2}}{2\iota_{12}}+\sum_{i=1}^{N}\frac{\mathrm{Tr}({\tilde{G}}_{xi}^{⊤}{\tilde{G}}_{xi})}{2\iota_{13}}, \end{array}$$

where ${\tilde{\kappa }}_{i11}=\kappa_{i11}-{\hat{\kappa }}_{i11}$, ${\tilde{\kappa }}_{i12}=\kappa_{i12}-{\hat{\kappa }}_{i12}$ and ${\tilde{G}}_{xi}=G_{xi}-{\hat{G}}_{xi}$.

The time derivative of (30) is given as

(31) $$\begin{array}{ccc} \dot{V}\; & \leq & \;e^{⊤}Le-e^{⊤}L^{2}\tilde{z}+2{\tilde{z}}^{⊤}\left(\Psi -\varphi \varphi^{⊤}\right)e-{\tilde{z}}^{⊤}\bar{L}\tilde{z}+\sum_{i=1}^{N}e_{i}^{⊤}F_{xi}\\ & & \;+\sum_{i=1}^{N}e_{i}^{⊤}\left({\tilde{G}}_{xi}+{\hat{G}}_{xi}\right)u_{xi}-\sum_{i=1}^{N}e_{i}^{⊤}{\dot{h}}_{i2}+\sum_{i=1}^{N}e_{i}^{⊤}\Delta_{xi}\\ & & \;+\sum_{i=1}^{N}\frac{{\tilde{\kappa }}_{i11}{\dot{\tilde{\kappa }}}_{i11}}{\iota_{11}}+\sum_{i=1}^{N}\frac{{\tilde{\kappa }}_{i12}{\dot{\tilde{\kappa }}}_{i12}}{\iota_{12}}+\sum_{i=1}^{N}\frac{\mathrm{Tr}({\tilde{G}}_{xi}^{⊤}{\dot{\tilde{G}}}_{xi})}{\iota_{13}}. \end{array}$$

Substituting the control input (26) into (31), we can obtain

(32) $$\begin{array}{ccc} \dot{V}\; & \leq & \;e^{⊤}Le-e^{⊤}L^{2}\tilde{z}+2{\tilde{z}}^{⊤}\left(\Psi -\varphi \varphi^{⊤}\right)e-{\tilde{z}}^{⊤}\bar{L}\tilde{z}-\sum_{i=1}^{N}\delta_{i}e_{i}^{⊤}e_{i}\\ & & \;+\sum_{i=1}^{N}e_{i}^{⊤}F_{xi}-\sum_{i=1}^{N}\frac{e_{i}^{⊤}e_{i}{\hat{\kappa }}_{i11}\omega_{i1}^{⊤}\omega_{i1}}{2\varsigma_{i1}^{2}}-\sum_{i=1}^{N}\frac{e_{i}^{⊤}e_{i}{\hat{\kappa }}_{i12}}{2\varsigma_{i2}^{2}}\\ & & \;+\sum_{i=1}^{N}e_{i}^{⊤}{\tilde{G}}_{xi}u_{xi}+\sum_{i=1}^{N}e_{i}^{⊤}\Delta_{xi}+\sum_{i=1}^{N}\frac{{\tilde{\kappa }}_{i11}{\dot{\tilde{\kappa }}}_{i11}}{\iota_{11}}\\ & & \;+\sum_{i=1}^{N}\frac{{\tilde{\kappa }}_{i12}{\dot{\tilde{\kappa }}}_{i12}}{\iota_{12}}+\sum_{i=1}^{N}\frac{\mathrm{Tr}({\tilde{G}}_{xi}^{⊤}{\dot{\tilde{G}}}_{xi})}{\iota_{13}}. \end{array}$$

Then, the RBFNN is used to approximate the unknown nonlinear function $F_{xi}$, which is shown as

(33) $$\begin{array}{c} F_{xi}=\theta_{i1}^{*⊤}\omega_{i1}+\epsilon_{i1}, \end{array}$$

where $∥\epsilon_{i1}∥\leq {\bar{\epsilon }}_{i1}$.

In terms of Young’s inequality, we can obtain

(34) $$\begin{array}{cc} e_{i}^{⊤}\theta_{i1}^{*⊤}\omega_{i1} & \leq \frac{e_{i}^{⊤}e_{i}\kappa_{i11}\omega_{i1}^{⊤}\omega_{i1}}{2\varsigma_{i1}^{2}}+\frac{\varsigma_{i1}^{2}}{2}, \end{array}$$

(35) $$\begin{array}{cc} e_{i}^{⊤}\left(\epsilon_{i1}+\Delta_{xi}\right) & \leq \frac{e_{i}^{⊤}e_{i}\kappa_{i12}}{2\varsigma_{i2}^{2}}+\frac{\varsigma_{i2}^{2}}{2}, \end{array}$$

where $\kappa_{i11}=\theta_{i1}^{*⊤}\theta_{i1}^{*}$ and $\kappa_{i12}={\left({\bar{\epsilon }}_{i1}+{\bar{\Delta }}_{xi}\right)}^{⊤}\left({\bar{\epsilon }}_{i1}+{\bar{\Delta }}_{xi}\right)$.

Substituting (34) and (35) into (32), we can obtain

(36) $$\begin{array}{ccc} \dot{V}\; & \leq & \;e^{⊤}Le-e^{⊤}L^{2}\tilde{z}+2{\tilde{z}}^{⊤}\left(\Psi -\varphi \varphi^{⊤}\right)e-{\tilde{z}}^{⊤}\bar{L}\tilde{z}-\sum_{i=1}^{N}\delta_{i}e_{i}^{⊤}e_{i}\\ & & \;+\sum_{i=1}^{N}\frac{e_{i}^{⊤}e_{i}\kappa_{i11}\omega_{i1}^{⊤}\omega_{i1}}{2\varsigma_{i1}^{2}}+\sum_{i=1}^{N}\frac{e_{i}^{⊤}e_{i}\kappa_{i12}}{2\varsigma_{i2}^{2}}-\sum_{i=1}^{N}\frac{e_{i}^{⊤}e_{i}{\hat{\kappa }}_{i11}\omega_{i1}^{⊤}\omega_{i1}}{2\varsigma_{i1}^{2}}\\ & & \;-\sum_{i=1}^{N}\frac{e_{i}^{⊤}e_{i}{\hat{\kappa }}_{i12}}{2\varsigma_{i2}^{2}}-\sum_{i=1}^{N}{\tilde{\kappa }}_{i1}\left(-k_{11}{\hat{\kappa }}_{i11}+\frac{e_{i}^{⊤}e_{i}\omega_{i1}^{⊤}\omega_{i1}}{2\varsigma_{i1}^{2}}\right)\\ & & \;+\sum_{i=1}^{N}e_{i}^{⊤}{\tilde{G}}_{xi}u_{xi}-\sum_{i=1}^{N}{\tilde{\kappa }}_{i2}\left(-k_{12}{\hat{\kappa }}_{i12}+\frac{e_{i}^{⊤}e_{i}}{2\varsigma_{i2}^{2}}\right)\\ & & \;-\sum_{i=1}^{N}\mathrm{Tr}\left({\overset{˜}{G}}_{xi}^{⊤}\left(-k_{13}{\hat{G}}_{xi}+e_{i}u_{xi}\right)\right)+\sum_{i=1}^{N}\frac{\varsigma_{i1}^{2}}{2}+\sum_{i=1}^{N}\frac{\varsigma_{i2}^{2}}{2}\\ \; & \leq & \;e^{T}Le-e^{T}L^{2}\tilde{z}+2{\tilde{z}}^{⊤}\left(\Psi -\varphi \varphi^{⊤}\right)e-{\tilde{z}}^{⊤}\bar{L}\tilde{z}-\sum_{i=1}^{N}\delta_{i}e_{i}^{⊤}e_{i}\\ & & \;-\sum_{i=1}^{N}\frac{k_{11}}{2}{\tilde{\kappa }}_{i11}^{2}-\sum_{i=1}^{N}\frac{k_{12}}{2}{\tilde{\kappa }}_{i12}^{2}-\sum_{i=1}^{N}\frac{k_{13}}{2}{∥{\tilde{G}}_{xi}∥}_{F}^{2}\\ & & \;+\sum_{i=1}^{N}\frac{k_{11}}{2}\kappa_{i11}^{2}+\sum_{i=1}^{N}\frac{k_{12}}{2}\kappa_{i12}^{2}+\sum_{i=1}^{N}\frac{k_{13}}{2}{∥G_{xi}∥}_{F}^{2}\\ & & \;+\sum_{i=1}^{N}\frac{\varsigma_{i1}^{2}}{2}+\sum_{i=1}^{N}\frac{\varsigma_{i2}^{2}}{2}. \end{array}$$

According to Lemma 1, we can obtain

(37) $$\begin{array}{c} -\tilde{z}\bar{L}\tilde{z}\leq -\frac{\lambda_{2}\left(\bar{L}\right)}{N}{∥\tilde{z}∥}^{2}. \end{array}$$

In terms of Young’s inequality, we can obtain

(38) $$\begin{array}{cc} -e^{⊤}L^{2}\tilde{z} & \leq \lambda_{\max}^{2}\left(L\right)∥e∥∥\tilde{z}∥\leq \frac{N\lambda_{\max}^{4}\left(L\right)}{\lambda_{2}\left(\bar{L}\right)}{∥e∥}^{2}+\frac{\lambda_{2}\left(\bar{L}\right)}{4N}{∥\tilde{z}∥}^{2}, \end{array}$$

(39) $$\begin{array}{cc} 2{\tilde{z}}^{⊤}\left(\Psi -\varphi \varphi^{⊤}\right)e & \leq 2∥e∥∥\tilde{z}∥\leq \frac{4N}{\lambda_{2}\left(\bar{L}\right)}{∥e∥}^{2}+\frac{\lambda_{2}\left(\bar{L}\right)}{4N}{∥\tilde{z}∥}^{2}. \end{array}$$

Substituting (37)–(39) into (36) yields that

(40) $$\begin{array}{ccc} \dot{V}\; & \leq & \;-\left(\delta -\lambda_{\max}\left(L\right)-\frac{N(\lambda_{\max}^{4}\left(L\right)+4)}{\lambda_{2}\left(\bar{L}\right)}\right){∥e∥}^{2}-\frac{\lambda_{2}\left(\bar{L}\right)}{2N}{∥\tilde{z}∥}^{2}\\ & & \;-\sum_{i=1}^{N}\frac{k_{11}}{2}{\tilde{\kappa }}_{i11}^{2}-\sum_{i=1}^{N}\frac{k_{12}}{2}{\tilde{\kappa }}_{i12}^{2}-\sum_{i=1}^{N}\frac{k_{13}}{2}{∥{\tilde{G}}_{xi}∥}_{F}^{2}+\sum_{i=1}^{N}\frac{k_{11}}{2}\kappa_{i11}^{2}\\ & & \;+\sum_{i=1}^{N}\frac{k_{12}}{2}\kappa_{i12}^{2}+\sum_{i=1}^{N}\frac{k_{13}}{2}{∥G_{xi}∥}_{F}^{2}+\sum_{i=1}^{N}\frac{\varsigma_{i1}^{2}}{2}+\sum_{i=1}^{N}\frac{\varsigma_{i1}^{2}}{2}\\ \; & \leq & \;-\varrho V+\mu , \end{array}$$

where $\delta =\min\{\delta_{1},\delta_{2},\ldots ,\delta_{N}\}$, $\varrho =\min\{2\left(\delta -\lambda_{\max}\left(L\right)-\frac{N(\lambda_{\max}^{4}\left(L\right)+4)}{\lambda_{2}\left(\bar{L}\right)}\right),\frac{\lambda_{2}\left(\bar{L}\right)}{2N},\iota_{11}k_{11},\iota_{12}k_{12},\iota_{13}k_{13}\}>0$, $\mu =\frac{1}{2}\sum_{i=1}^{N}(k_{11}\kappa_{i11}^{2}+k_{12}\kappa_{i12}^{2}+k_{13}∥G_{xi}{∥}_{F}^{2}+\varsigma_{i1}^{2}+\varsigma_{i2}^{2})$.

In terms of the boundedness theorem, the time-varying formation errors $\tilde{z}$ and *e* are uniformly ultimately bounded. Recalling the definition of ${\tilde{z}}_{i}$, $s_{i}$ and $e_{i}$, we can conclude that $s_{i}$ is uniformly ultimately bounded. According to the control objective, the time-varying formation of the HMASs in the XY plane is achieved. This completes the proof. □

**Remark** **3.** *The time-varying formation feasibility condition $h_{i2}-{\dot{h}}_{i1}=0$ indicates that there exists a constraint on the formation that can be achieved. Due to their dynamic limitations, the HMASs are unable to achieve any formations. Therefore, it is necessary to judge whether the feasibility condition is met when a formation is given. If the condition is met, the pre-designed time-varying formation can be achieved.*

## 4. Decentralized Formation Controller Design and Performance Analysis

In this section, a decentralized formation controller is designed to achieve the height tracking control of UAVs in the *Z* axis.

### 4.1. Decentralized Formation Controller Design

The altitude error system is defined as

(41) $$\begin{array}{cc} e_{izp} & =p_{aiz}-c_{ip}, \end{array}$$

(42) $$\begin{array}{cc} e_{izv} & =v_{aiz}-c_{iv}, \end{array}$$

where $c_{ip}$ is the desired position signal and $c_{iv}$ is the desired velocity signal.

The formation tracking control input and adaptive laws are designed as

(43) $$\begin{array}{ccc} u_{aiz}\; & = & \;{\hat{G}}_{aiz}^{-1}\left(-\sigma_{i}e_{i\zeta }-\frac{e_{i\zeta }{\hat{\kappa }}_{i21}\omega_{i2}^{⊤}\omega_{i2}}{2\varsigma_{i3}^{2}}-\frac{e_{i\zeta }{\hat{\kappa }}_{i22}}{2\varsigma_{i4}^{2}}+{\dot{c}}_{iv}-k_{i\zeta }e_{izv}\right), \end{array}$$

(44) $$\begin{array}{ccc} {\dot{\hat{\kappa }}}_{i21}\; & = & \;\iota_{21}\left(-k_{21}{\hat{\kappa }}_{i21}+\frac{e_{i\zeta }^{⊤}e_{i\zeta }\omega_{i2}^{⊤}\omega_{i2}}{2\varsigma_{i3}^{2}}\right), \end{array}$$

(45) $$\begin{array}{ccc} {\dot{\hat{\kappa }}}_{i22}\; & = & \;\iota_{22}\left(-k_{22}{\hat{\kappa }}_{i22}+\frac{e_{i\zeta }^{⊤}e_{i\zeta }}{2\varsigma_{i4}^{2}}\right), \end{array}$$

(46) $$\begin{array}{ccc} {\dot{\hat{G}}}_{aiz}\; & = & \;{\mathrm{Proj}}_{\left[{\underline{G}}_{aiz},{\bar{G}}_{aiz}\right]}\left\{S\right\}=\left\{\begin{array}{cc} 0, & \mathrm{if}\;{\hat{G}}_{aiz}={\bar{G}}_{aiz}\;\mathrm{and}\;S\geq 0\;\\ & \mathrm{or}\;{\hat{G}}_{aiz}={\underline{G}}_{aiz}\;\mathrm{and}\;S\leq 0,\\ S, & \mathrm{otherwise}, \end{array}\right. \end{array}$$

where $S=\iota_{23}\left(-k_{23}{\hat{G}}_{aiz}+e_{i\zeta }u_{aiz}\right)$, ${\underline{G}}_{aiz}$ and ${\bar{G}}_{aiz}$ are the lower bound and upper bound of the parameter $G_{xi}$, respectively, where $\sigma_{i}$, $k_{21}$, $k_{22}$, $k_{23}$, $k_{i\zeta }$, $\iota_{21}$, $\iota_{22}$, $\iota_{23}$, $\varsigma_{i3}$, $\varsigma_{i4}$ are positive parameters to be designed.

### 4.2. Performance Analysis

The performance of the altitude error system is given as follows.

**Theorem** **2.** *Consider the UAVs’ altitude control system (16). Suppose that Assumptions 1–3 hold and the feasibility condition $c_{iv}-{\dot{c}}_{ip}=0$ is satisfied, the formation tracking control scheme is designed as (43) and the adaptive laws are developed as (44)–(46), then the UAVs’ altitude trajectory can track the reference signal and the tracking error $e_{izp}$ is uniformly ultimately bounded.*

**Proof** **of Theorem 2.** If the feasibility condition $c_{iv}-{\dot{c}}_{ip}=0$ is satisfied, then the time derivative of the altitude error system (41) and (42) can be written as

(47) $$\begin{array}{cc} {\dot{e}}_{izp} & =e_{izv}, \end{array}$$

(48) $$\begin{array}{cc} {\dot{e}}_{izv} & =F_{aiz}+G_{aiz}u_{aiz}+\Delta_{aiz}-{\dot{c}}_{iv}. \end{array}$$

In the system (47), $e_{izv}$ can be regarded as the virtual control input. By designing the virtual control input $\zeta_{i}=-k_{i\zeta }e_{izp}$, the stability of the system (47) can be ensured.

Consider a positive Lyapunov function as

(49) $$\begin{array}{ccc} V_{izp}\; & = & \;\frac{1}{2}e_{izp}^{⊤}e_{izp}. \end{array}$$

The time derivative of (50) is given by

(50) $$\begin{array}{ccc} {\dot{V}}_{izp}\; & = & \;-k_{i\zeta }e_{izp}^{⊤}e_{izp}. \end{array}$$

Define a new error as

(51) $$\begin{array}{c} e_{i\zeta }=e_{izv}-\zeta_{i}. \end{array}$$

Taking the time derivative of (51) and substituting the control input (43) into it, we can obtain

(52) $$\begin{array}{ccc} {\dot{e}}_{i\zeta }\; & = & \;-\sigma_{i}e_{i\zeta }+F_{aiz}+\Delta_{aiz}+{\tilde{G}}_{aiz}u_{aiz}-\frac{e_{i\zeta }{\hat{\kappa }}_{i21}\omega_{i2}^{⊤}\omega_{i2}}{2\varsigma_{i3}^{2}}-\frac{e_{i\zeta }{\hat{\kappa }}_{i22}}{2\varsigma_{i4}^{2}}. \end{array}$$

Consider the following Lyapunov candidate function

(53) $$\begin{array}{ccc} V_{z}\; & = & \;\sum_{i=1}^{N}V_{iz}, \end{array}$$

where $V_{iz}$ is given as

(54) $$\begin{array}{ccc} V_{iz}\; & = & \;\frac{1}{2}e_{izp}^{⊤}e_{izp}+\frac{1}{2}e_{i\zeta }^{⊤}e_{i\zeta }+\frac{{\tilde{\kappa }}_{i21}^{2}}{2\iota_{21}}+\frac{{\tilde{\kappa }}_{i22}^{2}}{2\iota_{22}}+\frac{\mathrm{Tr}({\tilde{G}}_{aiz}^{⊤}{\tilde{G}}_{aiz})}{2\iota_{23}}, \end{array}$$

with ${\tilde{\kappa }}_{i21}=\kappa_{i21}-{\hat{\kappa }}_{i21}$, ${\tilde{\kappa }}_{i22}=\kappa_{i22}-{\hat{\kappa }}_{i22}$ and ${\tilde{G}}_{aiz}=G_{aiz}-{\hat{G}}_{aiz}$.

Taking the time derivative of (54) yields that

(55) $$\begin{array}{ccc} {\dot{V}}_{iz}\; & \leq & \;-k_{i\zeta }e_{izp}^{⊤}e_{izp}-\sigma_{i}e_{i\zeta }^{⊤}e_{i\zeta }+e_{i\zeta }^{⊤}F_{aiz}+e_{i\zeta }^{⊤}\Delta_{aiz}\\ & & \;+e_{i\zeta }^{⊤}{\tilde{G}}_{aiz}u_{aiz}-\frac{e_{i\zeta }^{⊤}e_{i\zeta }{\hat{\kappa }}_{i21}\omega_{i2}^{⊤}\omega_{i2}}{2\varsigma_{i3}^{2}}-\frac{e_{i\zeta }^{⊤}e_{i\zeta }{\hat{\kappa }}_{i22}}{2\varsigma_{i4}^{2}}\\ & & \;+\frac{{\tilde{\kappa }}_{i21}{\dot{\tilde{\kappa }}}_{i21}}{\iota_{21}}+\frac{{\tilde{\kappa }}_{i22}{\dot{\tilde{\kappa }}}_{i22}}{\iota_{22}}+\frac{\mathrm{Tr}({\tilde{G}}_{aiz}^{⊤}{\dot{\tilde{G}}}_{aiz})}{\iota_{23}}. \end{array}$$

Similarly, the RBFNN is used to approximate the unknown nonlinear function $F_{aiz}$, which is shown as

(56) $$\begin{array}{ccc} F_{aiz}\; & = & \;\theta_{i2}^{*⊤}\omega_{i2}+\epsilon_{i2}, \end{array}$$

where $∥\epsilon_{i2}∥\leq {\bar{\epsilon }}_{i2}$.

In terms of Young’s inequality, one can obtain

(57) $$\begin{array}{cc} e_{i\zeta }^{⊤}\theta_{i2}^{*⊤}\omega_{i2} & \leq \frac{e_{i\zeta }^{⊤}e_{i\zeta }\kappa_{i21}\omega_{i2}^{⊤}\omega_{i2}}{2\varsigma_{i3}^{2}}+\frac{\varsigma_{i3}^{2}}{2}, \end{array}$$

(58) $$\begin{array}{cc} e_{i\zeta }^{⊤}\left(\epsilon_{i2}+\Delta_{aiz}\right) & \leq \frac{e_{i\zeta }^{⊤}e_{i\zeta }\kappa_{i22}}{2\varsigma_{i4}^{2}}+\frac{\varsigma_{i4}^{2}}{2}, \end{array}$$

where $\kappa_{i21}=\theta_{i2}^{*⊤}\theta_{i2}^{*}$ and $\kappa_{i22}={\left({\bar{\epsilon }}_{i2}+{\bar{\Delta }}_{aiz}\right)}^{⊤}\left({\bar{\epsilon }}_{i2}+{\bar{\Delta }}_{aiz}\right)$.

Substituting (57) and (58) into (55), we can obtain

(59) $$\begin{array}{ccc} {\dot{V}}_{iz}\; & \leq & \;-k_{i\zeta }e_{izp}^{⊤}e_{izp}-\sigma_{i}e_{i\zeta }^{⊤}e_{i\zeta }+\frac{e_{i\zeta }^{⊤}e_{i\zeta }\kappa_{i21}\omega_{i2}^{⊤}\omega_{i2}}{2\varsigma_{i3}^{2}}+\frac{e_{i\zeta }^{⊤}e_{i\zeta }\kappa_{i22}}{2\varsigma_{i4}^{2}}\\ & & \;-\frac{e_{i\zeta }^{⊤}e_{i\zeta }{\hat{\kappa }}_{i21}\omega_{i2}^{⊤}\omega_{i2}}{2\varsigma_{i3}^{2}}-\frac{e_{i\zeta }^{⊤}e_{i\zeta }{\hat{\kappa }}_{i22}}{2\varsigma_{i4}^{2}}-{\tilde{\kappa }}_{i22}\left(-k_{22}{\hat{\kappa }}_{i22}+\frac{e_{i\zeta }^{⊤}e_{i\zeta }}{2\varsigma_{i4}^{2}}\right)\\ & & \;-{\tilde{\kappa }}_{i21}\left(-k_{21}{\hat{\kappa }}_{i21}+\frac{e_{i\zeta }^{⊤}e_{i\zeta }\omega_{i2}^{⊤}\omega_{i2}}{2\varsigma_{i3}^{2}}\right)+e_{i\zeta }^{⊤}{\tilde{G}}_{aiz}u_{aiz}\\ & & \;-\mathrm{Tr}\left({\tilde{G}}_{aiz}^{⊤}\left(-k_{23}{\hat{G}}_{aiz}+e_{i\zeta }u_{aiz}\right)\right)+\frac{\varsigma_{i3}^{2}}{2}+\frac{\varsigma_{i4}^{2}}{2}\\ \; & \leq & \;-k_{i\zeta }e_{izp}^{⊤}e_{izp}-\sigma_{i}e_{i\zeta }^{⊤}e_{i\zeta }-\frac{k_{21}}{2}{\tilde{\kappa }}_{i21}^{2}-\frac{k_{22}}{2}{\tilde{\kappa }}_{i22}^{2}-\frac{k_{23}}{2}{∥{\tilde{G}}_{aiz}∥}_{F}^{2}\\ & & \;+\frac{k_{21}}{2}\kappa_{i21}^{2}+\frac{k_{22}}{2}\kappa_{i22}^{2}+\frac{k_{23}}{2}{∥G_{aiz}∥}_{F}^{2}+\frac{\varsigma_{i3}^{2}}{2}+\frac{\varsigma_{i4}^{2}}{2}\\ \; & \leq & \;-\vartheta_{iz}V_{iz}+\nu_{iz}, \end{array}$$

where $\vartheta_{iz}=\min\left\{2k_{i\zeta },2\sigma_{i},\iota_{21}k_{21},\iota_{22}k_{22},\iota_{23}k_{23}\right\}>0$, $\nu_{iz}=\frac{k_{21}}{2}\kappa_{i21}^{2}+\frac{k_{22}}{2}\kappa_{i22}^{2}+\frac{k_{23}}{2}{∥G_{aiz}∥}_{F}^{2}$ $+\frac{\varsigma_{i3}^{2}}{2}+\frac{\varsigma_{i4}^{2}}{2}$.

According to (59), the derivative of (53) can be obtained

(60) $$\begin{array}{ccc} {\dot{V}}_{z}\; & \leq & \;-\vartheta_{z}V_{z}+\nu_{z}, \end{array}$$

where $\vartheta_{z}=\min\left\{\vartheta_{iz}\right\}$, $\nu_{z}=\sum_{i=1}^{N}\nu_{iz}$.

In terms of the boundedness theorem, the solution of the closed-loop system is uniformly ultimately bounded. Recalling the definitions of $e_{izp}$ and $e_{i\zeta }$, it can be concluded that the altitude tracking error and velocity error of UAVs is uniformly ultimately bounded. According to control objective, the pre-defined time-varying formation h(t) is achieved for multiple UAVs and USVs. This completes the proof. □

**Remark** **4.** *For simplicity, it is assumed that the height of each UAV in the existing literature [20] and [38] is the same and time-invariant when achieving a formation. However, in practical applications, the height of UAVs may vary according to the tasks. For the altitude control system of UAVs, a decentralized tracking controller is designed to track the reference signal to meet the requirements of practical tasks in the presence of actuator faults, parameter uncertainties and external disturbances in this paper, which has more application value.*

## 5. Simulation Study

**Figure 4:**
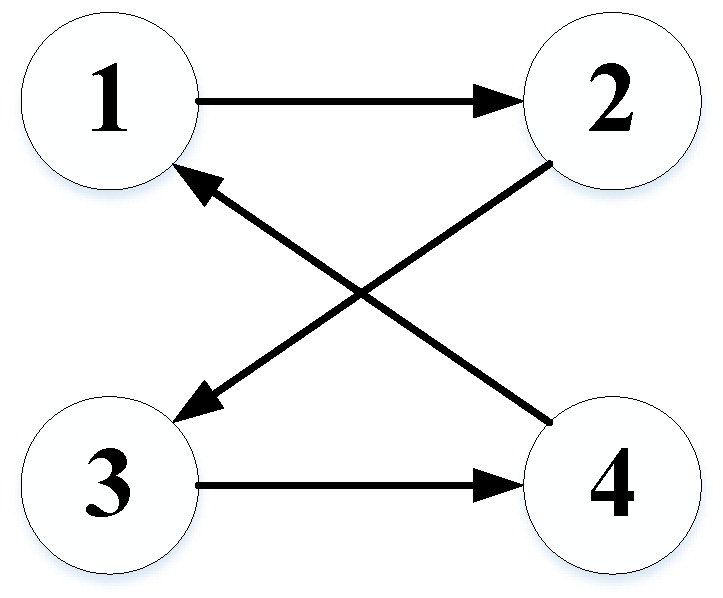
Directed communication topology.

In order to verify the effectiveness of the proposed fault-tolerant time-varying formation control scheme, a HMAS composed of two UAVs (i=1,2) and two USVs (i=3,4) is selected in this section. Table 1 shows the system parameters of UAVs and USVs. The directed communication topology is shown in Figure 4, in which the weights are selected as one.

The actuator faults of UAVs and USVs are given as

$$\begin{array}{ccc} & & \left\{\begin{array}{ccc} \rho_{a1}=\mathrm{diag}\left[1,1\right], & \;u_{a1b}={\left[0,0\right]}^{⊤}, & t<8\;s,\\ \rho_{a1}=\mathrm{diag}\left[0.9,0.7\right], & \;u_{a1b}={\left[0.15,0.3e^{-0.8(t-8)}\right]}^{⊤}, & t\geq 8\;s, \end{array}\right.\\ & & \left\{\begin{array}{cc} \rho_{a2}=\mathrm{diag}\left[1,1\right],\;\;\;u_{a2b}={\left[0,0\right]}^{⊤}, & \;\;t<12\;s,\\ \rho_{a2}=\mathrm{diag}\left[0.8,0.9\right],\;u_{a2b}={\left[0.2,0.1\right]}^{⊤}, & \;\;t\geq 12\;s, \end{array}\right.\\ & & \left\{\begin{array}{cc} \rho_{s3}=\mathrm{diag}\left[1,1\right],\;\;\;\;\;\;u_{s3b}={\left[0,0\right]}^{⊤}, & t<15\;s,\\ \rho_{s3}=\mathrm{diag}\left[0.8,0.7\right],\;u_{s3b}={\left[0.3,0.18\right]}^{⊤}, & t\geq 15\;s, \end{array}\right.\\ & & \left\{\begin{array}{cc} \rho_{s4}=\mathrm{diag}\left[1,1\right],\;\;\;\;\;\;\;\;u_{s4b}={\left[0,0\right]}^{⊤}, & \;\;t<18\;s,\\ \rho_{s4}=\mathrm{diag}\left[0.7,0.9\right],\;u_{s4b}={\left[0.2,0.4\right]}^{⊤}, & \;\;t\geq 18\;s. \end{array}\right. \end{array}$$

The actuator faults of UAVs in the *Z* axis are given as t<13s, $\rho_{a1z}=1$, $u_{a1bz}=0$; t≥13s, $\rho_{a1z}=0.9$, $u_{a1bz}=0.2$; t<10s, $\rho_{a2z}=1$, $u_{a2bz}=0$; t≥10s, $\rho_{a2z}=0.8$, $u_{a2bz}=0.1$.

The external disturbances are given as $\Delta_{a1xy}={\left[0.2\cos\left(0.5t\right),0.8\cos\left(t\right)\right]}^{⊤}$, $\Delta_{a1z}=0.3\cos\left(0.2t\right)$, $\Delta_{a2xy}={\left[0.6\sin\left(t\right),0.3\right]}^{⊤}$, $\Delta_{a2z}=0.5\sin\left(0.4t\right)$, $w_{d3xy}={\left[1.1\cos\left(0.5t\right),-0.2\sin\left(2t\right)\right]}^{⊤}$, $w_{d4xy}={\left[0.6,-0.2\cos\left(t\right)\right]}^{⊤}$. The desired time-varying formations are described as $h_{i1}={\left[3\cos\left(t+\left(i-1\right)\pi /2\right),3\sin\left(t+\left(i-1\right)\pi /2\right)\right]}^{⊤}$, $h_{i2}={\left[-3\sin\left(t+\left(i-1\right)\pi /2\right),3\cos\left(t+\left(i-1\right)\pi /2\right)\right]}^{⊤}$, i=1,2,3,4. $c_{ip}=0.1t$, $c_{iv}=0.1$, i=1,2. The initial values are chosen as $x_{11}\left(0\right)={\left[0.5,0.8,0\right]}^{⊤}$, $x_{21}\left(0\right)={\left[2.6,2.2,0\right]}^{⊤}$, $x_{31}\left(0\right)={\left[3.1,2.3\right]}^{⊤}$, $x_{41}\left(0\right)={\left[2.6,3.8\right]}^{⊤}$.

The performance of the proposed adaptive fault-tolerant time-varying formation tracking scheme is compared with the robust backstepping sliding-mode control scheme in [41]. To quantitatively evaluate the fault-tolerant time-varying formation tracking performance, *X*-axis position tracking error metric (XPTEM), *Y*-axis position tracking error metric (YPTEM), and *Z*-axis position tracking error metric (ZPTEM) are defined as

$$\mathrm{XPTEM}=\sqrt{\sum_{i=1}^{4}{\left|{\tilde{z}}_{ix}\right|}^{2}},\;\;\mathrm{YPTEM}=\sqrt{\sum_{i=1}^{4}{\left|{\tilde{z}}_{iy}\right|}^{2}},\;\;\mathrm{ZPTEM}=\sqrt{\sum_{i=1}^{2}{\left|{\tilde{e}}_{izp}\right|}^{2}}.$$

**Figure 5:**
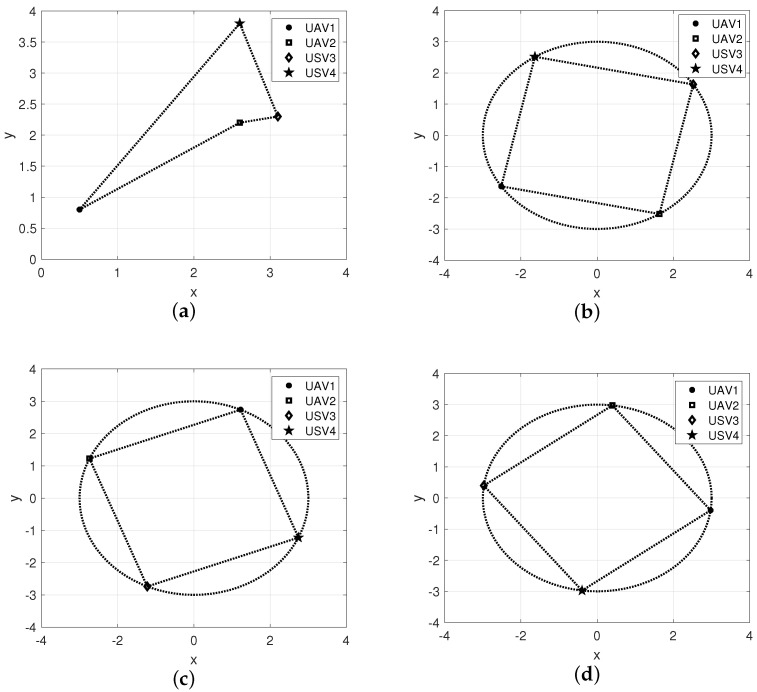
Position snapshots of the heterogeneous multi-agent system at different time instants. (**a**) t=0 s. (**b**) t=10 s. (**c**) t=20 s. (**d**) t=25 s.

**Figure 6:**
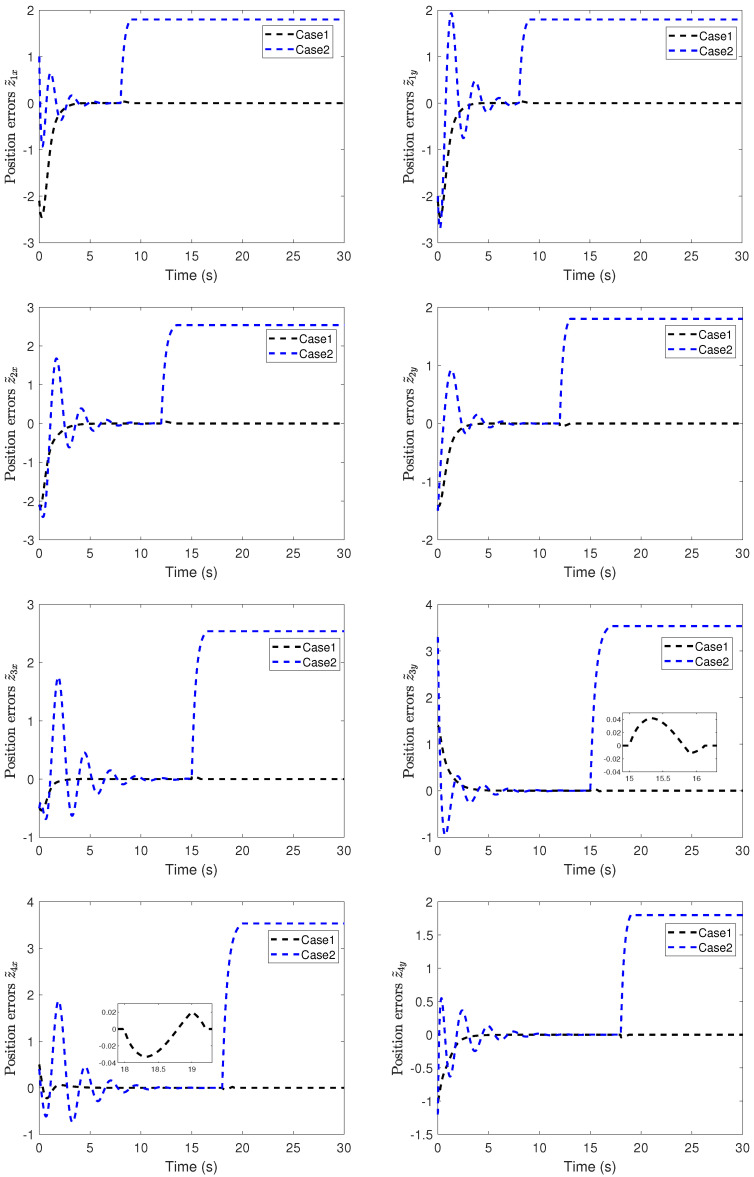
Position errors of time-varying formation in the XY plane (Case 1: proposed scheme; Case 2: scheme in [41]).

**Figure 7:**
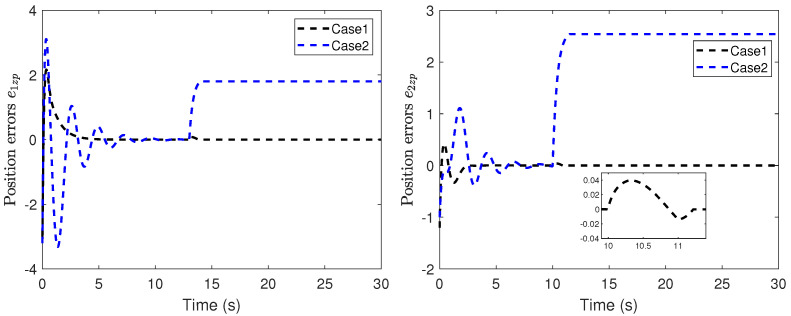
Altitude tracking error of UAVs (Case 1: proposed scheme; Case 2: scheme in [41]).

The position snapshots of the HMASs in the XY plane at different time instants is shown in Figure 5. The time-varying formation errors of all agents in the XY plane are depicted in Figure 6. The altitude tracking error of UAVs is shown in Figure 7.

**Figure 8:**
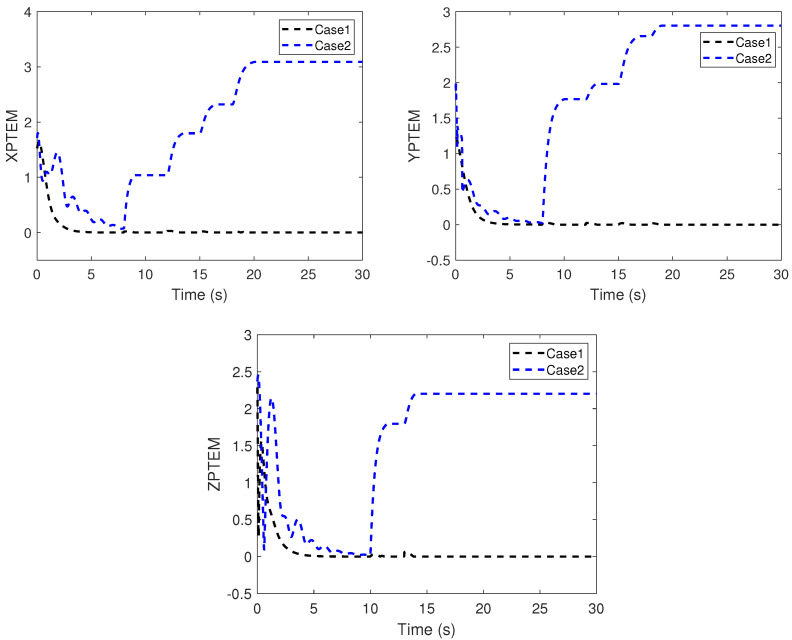
XPTEM, YPTEM, and ZPTEM.

From these curves, it can be seen that the tracking performance by the proposed control scheme is better than the robust backstepping sliding-mode control scheme in [41]. Furthermore, under the proposed control scheme, all tracking errors of the systems are uniformly ultimately bounded. From Figure 6 and Figure 7, it can be seen that the tracking errors under the scheme in [41] have sharp deviations from the small region containing zero when the actuator fault occurs. The position tracking error metrics are shown in Figure 8. It can be seen from these curves that the proposed control scheme has better performance than the scheme in [41]. From Figure 5, Figure 6, Figure 7 and Figure 8, we can know that the expected time-varying formation is achieved for the HMASs in the presence of actuator faults, parameter uncertainties and external disturbances. The effectiveness of the proposed fault-tolerant formation control scheme is verified by the simulation study.

## 6. Conclusions

In this paper, an adaptive fault-tolerant time-varying formation control scheme is designed for a heterogeneous multi-agent system composed of multiple UAVs and USVs with actuator faults, parameter uncertainties and external disturbances under directed communication topology. Based on the unified dynamic model of UAVs and USVs in the XY plane, a distributed fault-tolerant formation controller utilizing adaptive control and RBFNN is proposed. At the same time, a decentralized formation tracking controller is designed for the altitude control system of UAVs. Based on Lyapunov stability theory, the time-varying formation errors and tracking errors are uniformly ultimated bounded, and the pre-defined time-varying formation for multiple UAVs and USVs can be realized. Simulation results verify the effectiveness of the proposed scheme. Nevertheless, the communication topology is fixed in this study. Hence, a fault-tolerant formation control for multiple UAVs and USVs under switch topology will be investigated in our future works.

## Figures and Tables

**Table 1:** System parameters of UAVs and USVs.

Parameter	Value	Unit	Parameter	Value	Unit
$m_{ai}$	2	kg	$d_{\mu si}$	0.725	$\mathrm{kg}\cdot s^{-1}$
*g*	9.8	$m\cdot s^{-2}$	$d_{\nu si}$	0.89	$\mathrm{kg}\cdot s^{-1}$
$J_{ax},J_{ay},J_{az}$	1.5	$N\cdot s^{2}\cdot {\mathrm{rad}}^{-1}$	$d_{rsi}$	−1.9	$\mathrm{kg}\cdot m^{-2}\cdot s^{-1}$
$d_{ix},d_{iy},d_{iz}$	0.012	$N\cdot s\cdot {\mathrm{rad}}^{-1}$	$d_{\mu si1}$	−1.33	$\mathrm{kg}\cdot s^{-1}$
$m_{\mu si}$	25.8	kg	$d_{\nu si1}$	−36.47	$\mathrm{kg}\cdot s^{-1}$
$m_{\nu si}$	33.8	kg	$d_{rsi1}$	−0.75	$\mathrm{kg}\cdot m^{-2}\cdot s^{-1}$
$m_{rsi}$	2.76	kg			

## Data Availability

Not applicable.
